# Posterior Capsular Release in Soft‐Tissue Balancing to Correct Valgus Deformity and Flexion Contracture During Total Knee Arthroplasty

**DOI:** 10.1002/atn2.70390

**Published:** 2026-11-05

**Authors:** Takehiko Sugita, Yasuhito Sogi, Naohisa Miyatake, Akira Sasaki, Satoshi Kodajima, Ko Hashimoto, Toshimi Aizawa

**Affiliations:** ^1^ Department of Orthopaedic Surgery Tohoku Orthopaedic and Dental Clinic Sendai‐city Japan; ^2^ Department of Orthopaedic Surgery Tohoku Kousai Hospital Sendai‐city Japan; ^3^ Department of Orthopaedic Surgery Tohoku University Graduate School of Medicine Sendai‐city Japan

## Abstract

Total knee arthroplasty for valgus knee deformities presents many challenging problems, especially in soft‐tissue handling, although many soft‐tissue balancing techniques have been described to date. Posterior capsular release, a soft‐tissue balancing technique used to correct both valgus deformity and flexion contracture during total knee arthroplasty, is described in this technical note. Because this procedure does not sacrifice important lateral stabilizers, varus instability in knee flexion can be avoided. Furthermore, the release is performed just posterior to the femoral condyle without the risk of damaging the lateral collateral ligament and peroneal nerve associated with the posterior capsular division technique. We recommend this procedure as the first‐choice strategy because additional procedures are required in some cases.

## Graphical abstract

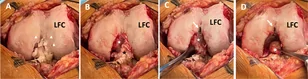

**VIDEO 1:** The video presents “posterior capsular release in soft‐tissue balancing to correct valgus deformity and flexion contracture during total knee arthroplasty.” We have no conflicts of interest to declare. After femoral and tibial bone cuts and posterior cruciate ligament resection, ligament balance and joint center gap are evaluated with the knee at 0° and 90° flexion and the patella reduced. If appropriate ligament balance and joint gap could not be achieved, posterior capsular release would be performed. First, a starting point for posterior capsular release is made at the posterior edge of the intercondylar fossa using an electrocautery, followed by a sharp raspatory. The starting point is <5 mm in length and progresses toward the lateral side of the fossa. Next, the middle part of the posterior capsule is completely released using a blunt dissector. The lateral part of the posterior capsule, which is approximately 1 cm proximal to the middle part of the attachment, is also released using a blunt dissector. At this point, ligament balance and joint center gap are re‐evaluated. Video content can be viewed at https://doi.org/10.1002/atn2.70390.

There have been many articles over the last three decades regarding the surgical approach, femoral and tibial bone cut, soft‐tissue handling, and component type during total knee arthroplasty (TKA) for valgus knee osteoarthritis.^1^, ^2^, ^3^, ^4^, ^5^, ^6^, ^7^, ^8^ Nevertheless, Alesi et al.^9^ stated in 2022 that there was no consensus regarding soft‐tissue structures that need to be addressed and the order of their release.

We focused on the idea of posterolateral capsular division for the correction of valgus deformity described by Beverland,^10^ Pagoti et al.,^11^ and Tucker et al.^12^ The most advantageous point of their idea is that both fixed flexion and valgus deformity can be corrected without sacrificing important lateral stabilizers, such as iliotibial band, lateral collateral ligament, and popliteus tendon.^12^ Beverland^10^ pointed out that contracture of the posterior capsule is greatest on the side of the deformity. In addition, Kinoshita et al.^13^ clarified the efficacy of posterior capsular release at the femoral attachment for flexion contractures during TKA. They showed that the efficacy of the release of the middle part of the posterior capsule was greater than that of the lateral and medial parts. Based on the above‐described ideas, we usually release the middle and lateral parts of the femoral attachment of the posterior capsule to correct valgus and flexion deformities during TKA for valgus knee osteoarthritis.

## SURGICAL TECHNIQUE

### Patient Positioning and Anesthesia

Lumbar spinal and general anesthesia are usually administered. After the induction of general anesthesia in the supine position, a tourniquet is secured around the upper thigh. The leg is prepared and draped in a sterile manner. The tourniquet is inflated to 250 mmHg.

### TKA Procedures

We usually apply a posterior cruciate ligament‐substituting (PS) or constrained PS Persona prosthesis (ZimmerBiomet, Warsaw, IN). With the patient in a supine position, a midline longitudinal skin incision and standard medial parapatellar arthrotomy are performed. First, the osteophytes formed at the front of the intercondylar fossa are removed to observe the femoral attachments of the anterior and posterior cruciate ligaments. These attachments are released from the intercondylar fossa using an electrocautery to expose the intercondylar fossa wall at the beginning of the operation. Before releasing the posterior cruciate ligament, its lateral margin is marked with an electrocautery on the femoral trochlea for use as a reference point to draw the anteroposterior axis of the femur (Whiteside's line) (Figure 1A‐D). Although the anterior cruciate ligament is thoroughly resected, the tibial attachment of the posterior cruciate ligament is preserved for use as a reference point for drawing the anteroposterior axis of the tibia (Akagi's line^14^) later. The femoral and tibial bones are cut using a mechanical alignment method. First, a distal femoral cut is made perpendicular to the femoral mechanical axis by using an accelerometer‐based portable navigation device (KneeAlign 2; OrthAlign, Aliso Viejo, CA)^15^ (Figure 2). When determining the distal and posterior femoral resection depths, the medial femoral condyle is used as a reference point, resulting in a minimal amount of bone being cut from the lateral femoral condyle. The rotational alignment of the femoral component is set at 3° of the externally rotated line relative to the clinical epicondylar line, that is, usually 5 ° to 7 ° of external rotation relative to the posterior condylar line. The tibial cut is also made perpendicular to the tibial mechanical axis with a posterior tibial slope of 3° using KneeAlign 2. The tibial resection depth is determined to be 9 mm distal to the intertubercle sulcus using a Persona tibial stylus (Figure 3), according to Sugita et al.^16^ and Kelmanovich et al.^17^ Rotational alignment of the tibial component is determined based on the intertubercle sulcus line and/or Akagi's line.^14^ After the femoral and tibial bone cuts, the posterior cruciate ligament is thoroughly resected. Subsequently, ligament balance (varus or valgus angle, degrees with positive values indicating varus balance) and joint center gap (mm) are evaluated with the knee at 0° and 90° flexion with the patella reduced using an offset‐type tensor (Offset Repo‐Tensor; ZimmerBiomet, Tokyo, Japan) described by Matsumoto et al.^18^ (Figure 4). If appropriate ligament balance and joint gap are not achieved, posterior capsular release would be performed (Video 1).

**FIGURE 1:**
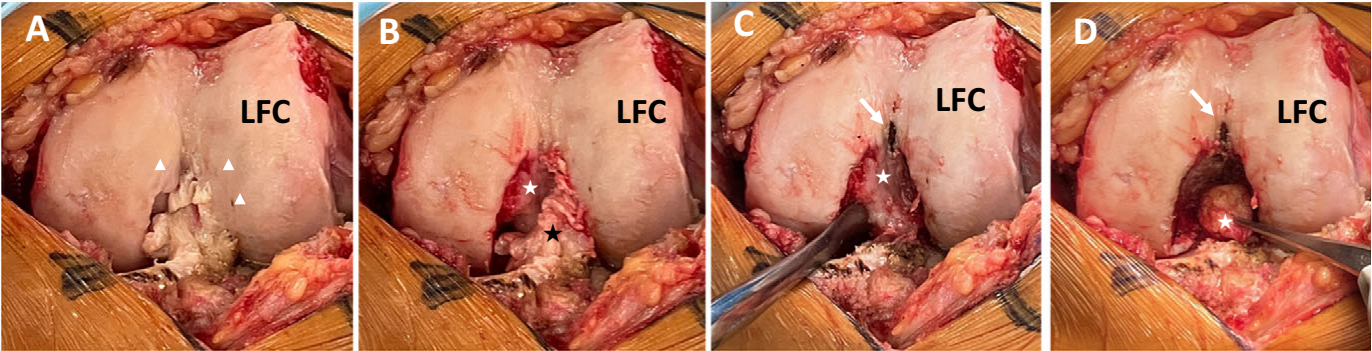
Osteophytes (arrowheads) are typically present at the front of the intercondylar fossa (A). The osteophytes are removed to observe the femoral attachment of the anterior (black asterisk) and posterior (white asterisk) cruciate ligaments (B). The anterior cruciate ligament is thoroughly resected. Before releasing the posterior cruciate ligament (white asterisk), the lateral margin of the posterior cruciate ligament is marked with an electrocautery on the femoral trochlea (white arrow) (C). The femoral attachment of the posterior cruciate ligament is released (white asterisk) from the intercondylar fossa using an electrocautery. The wall of the intercondylar fossa is clearly exposed (D). (LFC, lateral femoral condyle.)

**FIGURE 2:**
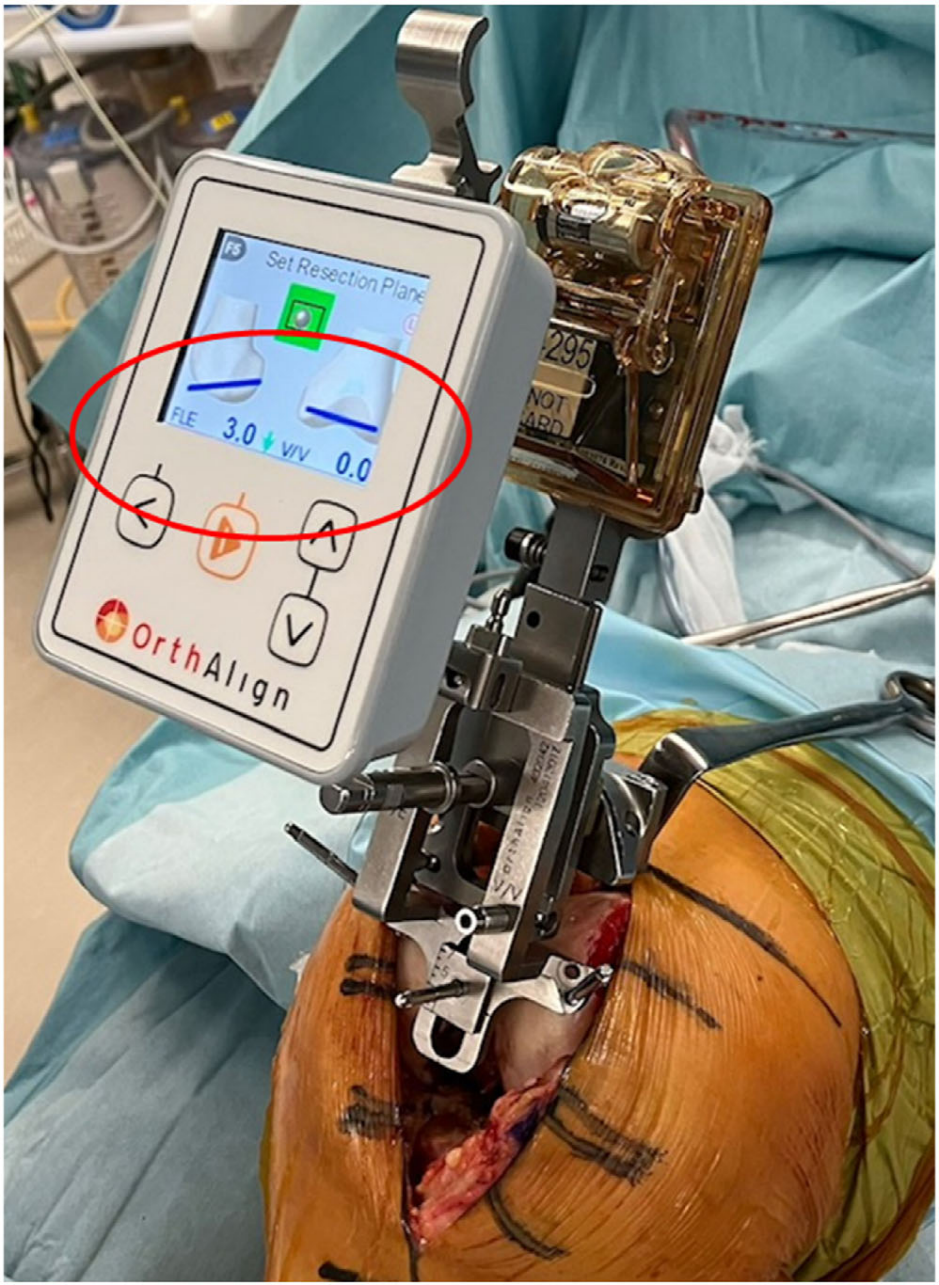
The distal femoral cut is set perpendicular and 3.0° flexed position to the femoral mechanical axis (red circle) using an accelerometer‐based, portable navigation device.

**FIGURE 3:**
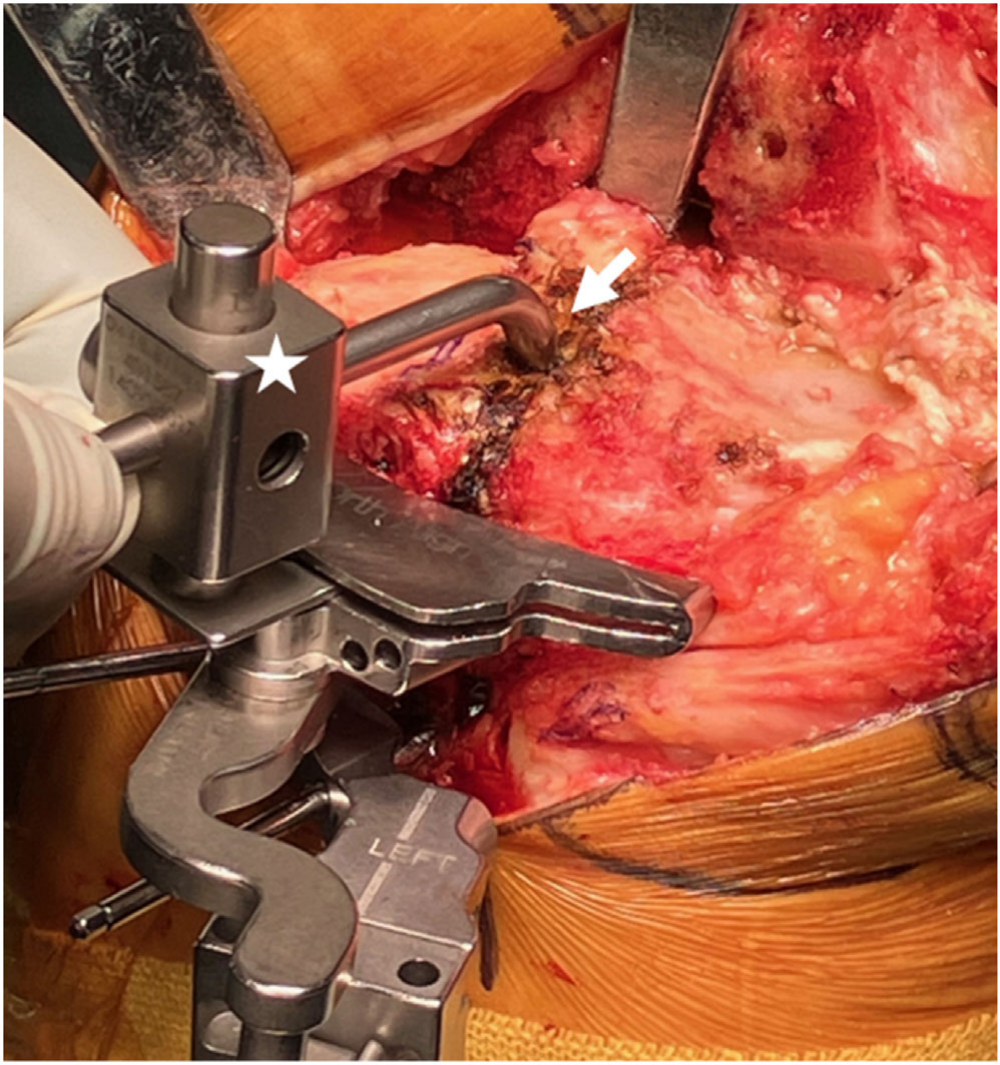
The tibial resection depth is set 9 mm distal to the intertubercle sulcus using Persona tibial stylus (white asterisk). The tip of the stylus touches the floor of the sulcus (white arrow).

**FIGURE 4:**
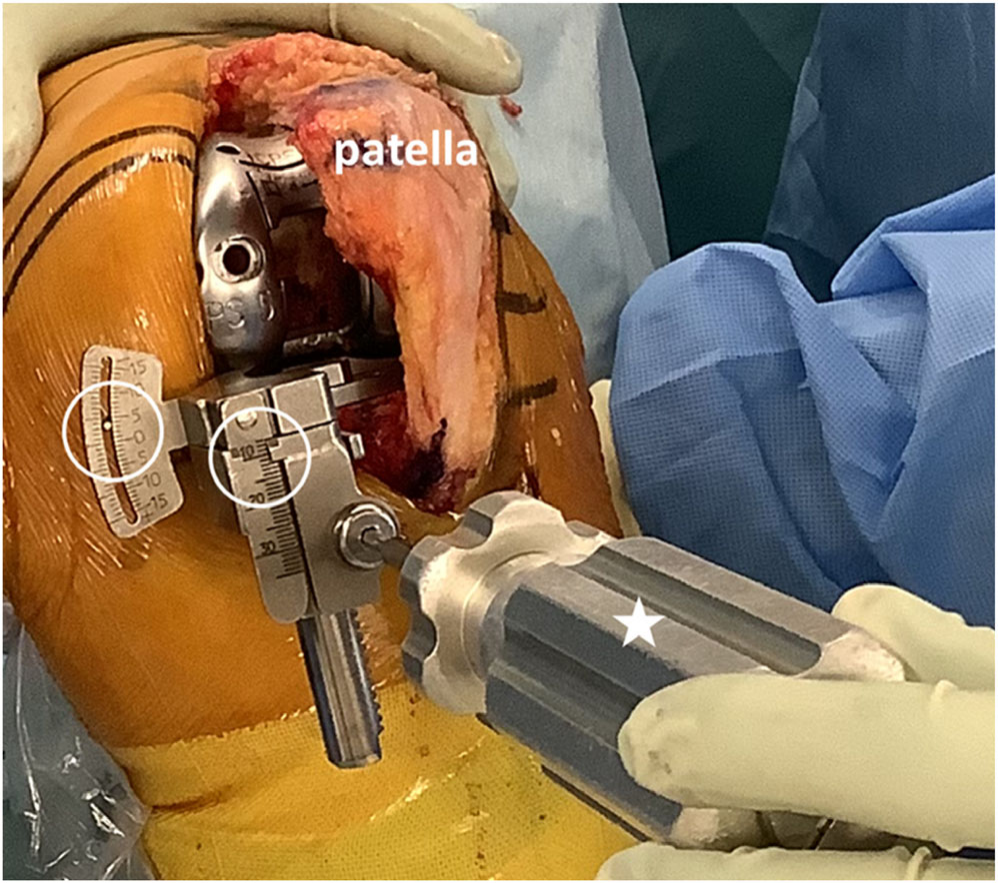
The ligament balance and joint center gap are measured with the knee at 0° and 90° flexion with the patella reduced using an offset‐type tensor. Joint distraction force is applied at 40 lb using a torque driver (white asterisk). White circles indicate that the ligament balance is 4° of valgus and joint center gap is 8 mm.

### Posterior Capsular Release

Before the posterior capsular release, it is important to completely remove the remnants of the femoral attachments of the anterior and posterior cruciate ligaments to clearly expose the upper and lateral walls of the intercondylar fossa. It is also important to resect the osteophytes and/or excessive bone at the posterolateral corner of the intercondylar fossa (Figure 5A and B). First, the starting point of the posterior capsular release is made at the posterior edge of the intercondylar fossa using an electrocautery, followed by a sharp raspatory (Figure 6A and B). The starting point is <5 mm in length and progresses toward the lateral side of the fossa. Next, the middle part of the posterior capsule is completely released using a blunt dissector. The lateral part of the posterior capsule, which attaches approximately 1 cm proximal to the middle part of the attachment, is released using a blunt dissector (Figure 7A and B). At this point, the ligament balance and joint center gap are re‐evaluated. Once appropriate ligament balance and joint gap are confirmed, the femoral and tibial components are fixed using bone cement, and a PS or constrained PS tibial surface is inserted. We usually use a constrained PS tibial surface when more than 3 mm of valgus instability remains with the knee extended while inserting the PS tibial surface trial.

**FIGURE 5:**
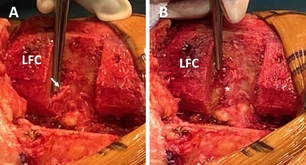
There exists osteophyte (white arrow) at the posterolateral corner of the intercondylar fossa (A). After removing the osteophyte, the posterior capsule (white asterisk) is clearly visible (B). (LFC, lateral femoral condyle.)

**FIGURE 6:**
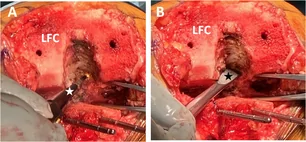
A starting point of the posterior capsular release is made at the posterior edge and lateral side of the intercondylar fossa using an electrocautery (white asterisk) (A) and then a sharp raspatory (black asterisk) (B). The starting point needs <5 mm length. (LFC, lateral femoral condyle.)

**FIGURE 7:**
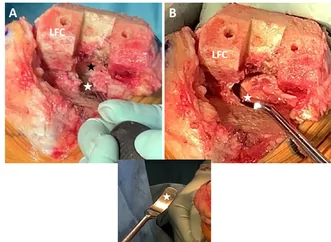
The posterior capsular release is performed using a blunt dissector (white asterisk). A black asterisk indicates the released middle part of the posterior capsule (A). Next, the lateral part of the posterior capsule is released using a blunt dissector (white asterisk) (B). (LFC, lateral femoral condyle.)

### Rehabilitation After Surgery

The patients begin using wheelchairs on postoperative day 1. Passive range‐of‐motion exercises with a continuous passive motion machine and lower‐extremity muscle training begin on postoperative day 2. Walking with the assistance of a walker is initiated on postoperative day 3 or 4.

## DISCUSSION

The advantages and limitations of this procedure are listed in Table 1. The main advantage is the ability to avoid releasing lateral stabilizing structures such as the lateral collateral ligament, popliteus tendon, and iliotibial tract. The target structures of release were the middle and lateral parts of the posterior capsule. Valgus deformities and flexion contractures could also be corrected using this procedure. Furthermore, this blunt release could allow for avoiding not only damage to popliteal neurovascular structures but also peroneal nerve injury^3^, ^5^ and lateral collateral ligament damage,^6^ which may be associated with horizontal posterolateral capsular division.^10^, ^11^, ^12^ One limitation is that further procedures, such as tibial recut, distal femoral recut, and horizontal cut of the tight anterior part of the iliotibial band, might be required to obtain an appropriate joint gap and ligament balance in certain knees. Another limitation is that releasing lateral stabilizing structures might be required for fixed severe valgus knees, such as Ranawat type III knees. Furthermore, robotic‐assisted TKA is reported as a safe and effective approach for patients with valgus deformity recently,^19^, ^20^ although this piece of evidence is based on short‐term follow‐up. Therefore, the proposed posterior capsular release method could be used as a first‐choice procedure.

**TABLE 1:** Advantages and Limitations of This Technique

**Advantages**	**Limitations**
1) Releasing lateral stabilizing structures, such as the lateral collateral ligament, popliteus tendon, and iliotibial tract, could be avoided because the target structures of release are only middle and lateral parts of the posterior capsule 2) Both valgus deformity and flexion contracture could be corrected by this procedure 3) This blunt release of the posterior capsule could allow for avoiding the risk of popliteal neurovascular damage, peroneal nerve injury, and lateral collateral ligament damage	1) Further procedures, such as tibial recut, distal femoral recut, and horizontal cut of tight anterior part of the iliotibial band, might be required to obtain an appropriate joint gap and ligament balance in certain knees 2) Releasing lateral stabilizing structures might be required for fixed severe valgus knees, such as Ranawat type III knees 3) Robotic‐assisted TKA is reported as a safe and effective approach for patients with valgus deformity

TKA, total knee arthroplasty.

A blunt dissector is mandatory to avoid damage to the popliteal neurovascular structures and the peroneal nerve. There are some pearls and pitfalls for blunt dissection of the posterior capsule (Table 2). First, it would be better to remove the osteophytes at the front of the intercondylar fossa and explore and release the femoral attachments of the anterior and posterior cruciate ligaments at the beginning of the operation to clearly expose the intercondylar fossa wall. Second, we recommend establishing a starting point for posterior capsular release at the posterior edge of the intercondylar fossa using an electrocautery, followed by a sharp raspatory. The starting point needs to be at least 5 mm in length for the insertion of a blunt dissector. Before establishing the starting point, the osteophytes or excessive bone at the posterolateral corner of the fossa should be removed. Finally, regarding the length of the posterior capsular release, approximately 3 and 4 cm length at the middle and lateral parts of the capsule, respectively, are recommended, according to Kinoshita et al.^13^ A potential pitfall is the inadvertent release of the femoral tendon origin of the lateral head of the gastrocnemius during releasing the lateral part of the posterior capsule because the clinical consequences of such detachment remain unclear.

**TABLE 2:** Pearls and Pitfalls for Blunt Dissection of the Posterior Capsule

**Pearls**	**Pitfalls**
1) Exposing the wall of the intercondylar fossa clearly is essential by removing osteophytes at the front of the intercondylar fossa and exploring and releasing the femoral attachments of the anterior and posterior cruciate ligaments at the beginning of the operation 2) Making a starting point of the posterior capsular release at the posterior edge of the intercondylar fossa using an electrocautery, followed by a sharp raspatory. The starting point requires a length of at least 5 mm for blunt dissector insertion 3) Removing osteophytes or excessive bone at the posterolateral corner of the fossa before making a starting point 4) Releasing the posterior capsule about 3 and 4 cm in length at the middle and lateral parts of the capsule, respectively	1) Releasing the femoral tendon origin of the lateral head of the gastrocnemius while releasing the lateral part of the posterior capsule

## DISCLOSURES

The authors (T.S., Y.S., N.M., A.S., S.K., K.H., T.A.) declare that they have no known competing financial interests or personal relationships that could have appeared to influence the work reported in this article.
